# Susceptibility of *ECE1* polymorphisms to Hirschsprung's disease in southern Chinese children

**DOI:** 10.3389/fped.2022.1056938

**Published:** 2022-12-22

**Authors:** Chaoting Lan, Yanqing Liu, Xiao Wu, Bingtong Wang, Songqing Xin, Qiuming He, Wei Zhong, Zipeng Liu

**Affiliations:** ^1^ Department of Pediatric Surgery, Guangzhou Institute of Pediatrics, Guangzhou Women and Children’s Medical Center, Guangzhou Medical University, Guangzhou, Guangdong, China; ^2^ Guangzhou Women and Children's Medical Center, Guangdong Provincial Clinical Research Center for Child Health, Guangzhou Medical University, Guangzhou, China; ^3^ Changan Hospital of Dongguan, Dongguan, China

**Keywords:** Hirschsprung's disease, Endothelin Converting Enzyme-1, single nucleotide polymorphism, epigenetic regulation, HSCR subtypes

## Abstract

**Background:**

Hirschsprung's disease (HSCR) is currently considered to be a congenital gastrointestinal malformation caused mainly by genetic factors. Endothelin Converting Enzyme-1 (*ECE1*) has been reported to be associated with HSCR. However, the relationship between *ECE1* single nucleotide polymorphism (SNP) rs169884 and HSCR in the southern Chinese population remains unknown.

**Methods:**

1,470 HSCR patients and 1,473 controls from a southern Chinese population were recruited. The intronic SNP rs169884 in *ECE1* was genotyped in all samples. We tested the association between rs169884 and HSCR under various genetic models. We also evaluated the effect of rs169884 on HSCR subtypes, including short-segment HSCR (S-HSCR), long-segment HSCR (L-HSCR) and total colonic aganglionosis (TCA). External epigenetic data were integrated to investigate the potential biological function of rs169884.

**Results:**

Chromatin states data from derived neuron cells or fetal colon tissue revealed that rs169884 might control *ECE1* expression through regulating its enhancer function. We did not find a significant association between rs169884 and HSCR. For HSCR subtypes, although no significant associations were detected between rs169884 and S-HSCR (*OR *= 1.00, *95% CI*: 0.89∼1.12, *P_adj _*= 0.77) or TCA (*OR *= 1.00, *95% CI*: 0.72∼1.38, *P_adj _*= 0.94), we found that rs169884 could increase the risk of L-HSCR (*OR* = 1.23, *95% CI* 1.02∼1.45, *P_adj _*= 0.024).

**Conclusion:**

These results suggested that rs169884 might play a regulatory role for *ECE1* expression and increase susceptibility of L-HSCR in southern Chinese children.

## Introduction

Hirschsprung's disease (HSCR) is a common gastrointestinal malformation worldwide (1). The abnormal development of neural crest cells (NCC) in embryo is the main cause of the disease (2). If NCC fail to colonize at the end of the digestive tract during development, functionally mature ganglia is lacking, resulting in intestinal lesions (3). The diseased bowel segment that lacks ganglia shows spastic stricture, causing abdominal distension and constipation (4). According to the current statistics, the incidence of HSCR has a difference in races and genders, with a global incidence of about 1 in 5,000 and a higher incidence of about 1.4 in 5,000 in Asia (5). The incidence of HSCR is 4 to 1 between males and females (6). Clinicians have classified HSCR into short-segment HSCR (S-HSCR), long-segment HSCR (L-HSCR) and total colonic aganglionosis (TCA), accounting for about 60%–80%, 10%–15% and 5% of HSCR cases, respectively (7). From a genetic perspective, only 10%–15% of HSCR patients have familial inheritance, while more than 70% are sporadic cases (8). Increasing studies have shown that HSCR is a highly polygenic disease. At present, nearly 20 genes (such as *RET*, *EDNRB*, *SOX10*, etc 9, 10). have been reported to be associated with HSCR. Among these genes, *GDNF*/*RET* comprise a signaling pathway which is a validated pathogenic mechanism underlying HSCR (11, 12). Abnormal signal transduction affect the differentiation, proliferation and migration of enteric neural crest cells (ENCCs), which leads to colon aganglionosis and onset of HSCR (3).

Endothelin Converting Enzyme-1 (*ECE1*), a peptide-chain endonuclease that transforms large endothelin (ET) into active ET *in vivo*, is a highly glycosylated glycoprotein that plays an extremely important role in the regulation of endothelin bioactivity (13). *ECE1* has been found to be widely expressed in intestinal tissues, controlling intestinal motor and secretory function by promoting the production of endothelin (14). More importantly, there is abundant *ECE1* in the endosome of intestinal myenteric neurons, which plays an important role in degrading some neuropeptides in the endosome, thereby regulating the transport of important receptors and signal transmission (15, 16). Further investigation revealed that *ECE1* knockout mouse models exhibited a phenotype of intestinal neuron depletion, suggesting an important role in neuronal development (17).

According to previous studies, *ECE1* is involved in the pathogenesis of HSCR (9, 18). Considering that rs169884 locates at a regulatory site in the genomic region of *ECE1*, we conducted a case–control study to explore the association between *ECE1* rs169884 polymorphism and HSCR susceptibility in Southern Chinese population.

## Materials and methods

### Study subjects

All participants in this study were recruited from Guangzhou Women and Children's Medical Center. Signed informed consent had been obtained from guardians of all participants prior to enrollment in the study. In the case group, patients were diagnosed with HSCR by histological examination of colon biopsies after surgical resection, and were classified into S-HSCR, L-HSCR, and TCA according to the length of the aganglionosis colon by pathologists. Control samples were obtained from patients without HSCR, enteritis, neurological disease or HSCR-related familial history based on the medical files at the time of enrollment. No environmental factors (e.g., toxic or drug exposure) that strongly associated with predisposition to HSCR have been identified in control samples from the medical records. Data of age, gender and ethnic were collected from the medical files for both HSCR and control samples. The significance test of differences in characteristics between cases and controls were analyzed with $\chi^{2}$ -test or Mann Whitney U Test based on data properties. All participants were unrelated Chinese Han. Ethical approval was obtained from the Institutional Review Board of Guangzhou Women and Children's Medical Center (Ethical Approval Number: 2018052406).

### Chromatin states data

The chromatin states were obtained from ROADMAP project (http://www.roadmapepigenomics.org). We selected data from cells/tissues related with HSCR: (1) H1 derived neuronal progenitor cultured cells; (2) H9 derived neuronal progenitor cultured cells; (3) H9 derived neuron cultured cells; (4) Fetal large intestine. The result was visualized using WashU Epigenome Browser (http://epigenomegateway.wustl.edu/browser/).

### SNP genotyping

Genomic DNA were extracted from venous blood of all samples. The SNP rs169884 was genotyped by Sequenom MassARRAY iPLEX Gold system (San Diego, CA, USA).

### Statistical analysis

The association test was performed using PLINK1.9 software (https://www.cog-genomics.org/plink/1.9/). The Hardy–Weinberg equilibrium (HWE) was calculated for this SNP in the control group and *P* > 0.05 was considered a good genotyping quality. Various genetic models were investigated, including the allelic model (ALLELIC), the 2df genotypic model (GENO), the additive model (ADD), the dominant model (DOM) and the recessive model (REC). Given the controls and HSCR cases, we used “–logistic” option to calculate the odds ratio (OR) and its significance for the rs169884 risk on HSCR. The age and gender were used as covariates to calculate the adjusted OR and *P* value. For the allelic model, we used “–assoc” option to run the 1df chi-square allelic test. Then we performed similar analysis for HSCR subtypes, i.e., S-HSCR, L-HSCR, and TCA, respectively.

## Results

### Regulatory potential of rs169884

**Figure 1:**
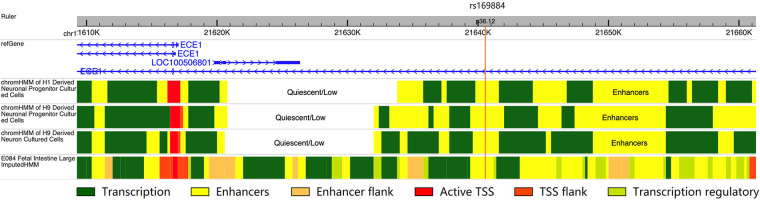
The chromatin states of the genomic region surrounding rs169884. The chromatin states were obtained from H1 or H9 derived neuronal progenitors, H9 derived neurons as well fetal large intestine. The genomic region nearby rs169884, which belongs to the *ECE1* intron, was enriched with enhancer signals across the selected cell types or tissue. The genomic version was hg19.

To investigate the potential regulatory function of rs169884, we collected data of chromatin states along the *ECE1* genomic region from three derived neuron-related cells and the fetal colon tissue (Figure 1). Interestingly, we noticed that the genomic region nearby rs169884 showed obvious enhancer signals in all the selected chromatin states data. This suggests that rs169884 might play a role for *ECE1* expression through regulating the enhancer function.

### Association of *ECE1* SNP with HSCR susceptibility

A total of 1,470 HSCR patients and 1,473 controls were included in the study. The clinical information of the participants was summarized in Table 1. The genotype frequency of rs169884 and the association analysis between rs169884 and HSCR were calculated, as shown in Table 2. As shown, a total of 1,398 cases and 1,464 controls were successfully genotyped. HWE was confirmed in the control group (*P* > 0.05). Association between rs169884 and HSCR was evaluated under the allelic model (ALLELIC), the 2df genotypic model (GENO), the additive model (ADD), the dominant model (DOM) and the recessive model (REC), respectively. Since we noticed significant difference for age and gender between the case and control group (Table 1), we further run the logistic regression adjusted for the two covariates. In all five genetic models, we could not verify a significant association between rs169884 and HSCR (Table 2).

**Table 1:** Clinical characteristics of the subjects.

Characteristic	Cases (*n* = 1,470)	Controls (*n* = 1,473)	*P*
Gender (Female/Male)	240/1230	458/1015	<0.01^a^
Age (month), mean ± SD	8.37 ± 20.50	18.61 ± 19.75	<0.01^b^
Ethnics	Chinese Han	Chinese Han	
Clinical manifestation (%)			
S-HSCR	1,034 (70.34%)	N/A	-
L-HSCR	295 (20.07%)	N/A	-
TCA	82 (5.58%)	N/A	-
TIA	3 (0.20%)	N/A	-
Unknown subtype	56 (3.81%)	N/A	-

^a^ $\chi^{2}$-test.

^b^ Mann Whitney U Test.

SD, standard deviation; NA, not available.

S-HSCR, short-segment HSCR; L-HSCR, long-segment HSCR; TCA, total colonic aganglionosis; TIA, Total intestine aganglionosis.

**Table 2:** Results of SNP rs160844 in a southern Chinese population of 1,470 cases and 1,473 controls.

CHR	SNP	BP	Gene	A1/A2	F_A	F_U	HWE	Model	AFF	UNAFF	OR (CI 0.95)	*P*	OR.adj	*P*.adj
1	rs169884	21640565	*ECE1*	T/C	0.39	0.38	0.287	ALLELIC T/C	1093/1703	1112/1816	1.05 (0.94–1.17)	0.39	-	-
								GENO	204/685/509	207/698/559	-	0.62	-	0.5
								ADD TT/TC/CC	204/685/509	207/698/559	1.05 (0.94–1.17)	0.38	1.07 (0.95–1.20)	0.26
								DOM (TT + TC)/CC	889/509	905/559	1.08 (0.93–1.25)	0.33	1.10 (0.94–1.29)	0.24
								REC TT/ (TC + CC)	204/1194	207/1257	1.04 (0.84–1.28)	0.73	1.07 (0.85–1.33)	0.58

CHR, chromosome; SNP, single-nucleotide polymorphism; BP, SNP position in genome (hg19); A1/A2, risk/protective allele; F_A/F_U, minor allele frequency of the SNP in cases and controls, respectively; HWE, *P* value of Hardy–Weinberg equilibrium test; Model, ALLELIC, GENO, ADD, DOM, and REC corresponds to allelic association, genotypic association (2df), additive dominant and recessive model, respectively; AFF, allele count in cases under different models; UNAFF, allele count in controls under different models; OR: odds ratio of the risk allele under different tests; CI, confidence interval; *P*, unadjusted *P* values under different tests; OR.adj, odds ratio under different tests adjusted for gender and age; *P*.adj, *P* value under different tests adjusted for gender and age.

### Stratification analysis of *ECE1* rs169884 with HSCR subtypes

Further stratification analyses were carried out to assess whether rs169884 is associated with HSCR subtypes (S-HSCR, L-HSCR, and TCA). The results revealed that rs169884 was significantly associated with L-HSCR (*OR *= 1.23, *95% CI*: 1.02∼1.48, *P_adj _*= 0.024). Evidence of association between rs169884 and S-HSCR or TCA is insufficient (*OR  *= 1.00, *95% CI*: 0.89∼1.12, *P_adj _*= 0.77; *OR  *= 1.00, *95% CI*: 0.72∼1.38, *P_adj _*= 0.94) (Table 3).

**Table 3:** Association between SNP rs169844 and different subtypes of HSCR.

CHR	SNP	BP	A1/A2	HSCR subtypes	AFF	UNAFF	OR (CI 0.95)	*P*	*P*.adj
1	rs169884	21640565	T/C	S-HCSR	742/1216	1112/1816	1.00 (0.89∼1.12)	0.97	0.77
				L-HSCR	240/322		1.23 (1.02∼1.48)	0.031	0.024
				TCA	59/101		1.00 (0.72∼1.38)	1.00	0.94

CHR, chromosome; SNP, single-nucleotide polymorphism; BP, SNP position in genome (hg19); A1/A2, risk/protective allele; AFF, risk/protective allele count in cases; UNAFF, risk/protective allele frequency in controls; OR, odds ratio of the risk allele by logistic regression; CI, confidence interval; *P*, unadjusted *P* values by logistic regression; *P*.adj, *P* values adjusted for gender and age by logistic regression.

## Discussion

As a congenital digestive defect, HSCR is a life-threatening disease. Although surgery can largely relieve a patient's condition, complications after surgery can be lifelong (19), and the suffering caused by the disease is enormous (20). However, the knowledge of the pathogenesis of HSCR is still very limited.

**Figure 2:**
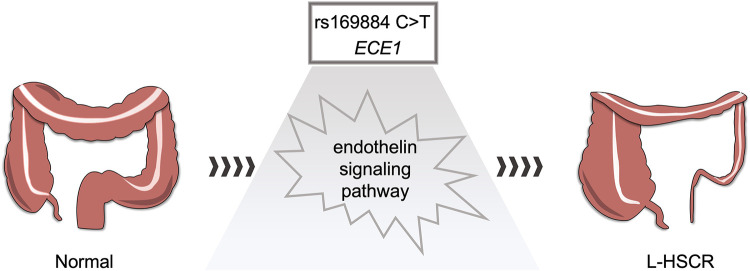
Hypothesis of rs169884 risk on L-HSCR. rs169884 may regulate the expression of *ECE1*, which further causes abnormal development of enteric nervous system through the endothelin signaling pathway.

In our study, we focused on the HSCR susceptibility of *ECE1* gene, a key component of endothelin signaling pathway. This pathway is critical in regulating the development of the cardiovascular system, kidney, and pulmonary processes, as well as vertebrate-specific neural crest cell populations and their derivatives, especially at an early stage (21). The endothelin system has been found to be essential in the development of neural crest-derived tissues, including intestinal neurons (22). At the embryonic stage, endothelin signaling is required for the migration of enteric neural progenitors from the foregut to the hindgut. On the other hand, lack of appropriate endothelin signaling in mice and human leads to HSCR phenotype (23). At present, several members in the endothelin signaling pathway have been proved to be closely related to the development of Hirschsprung's disease (22, 24, 25). *ECE1*, an important member in endothelin signaling pathway, can regulate neurokinin 1 receptor (*NK1R*) trafficking and signal transduction in endosomes of myoenteric neurons, attenuating *NK1R*-mediated *ERK1/2* activation in myoenteric neurons by promoting *NK1R* re-sensitivity, leading to intestinal neuron depletion (26). In addition, loss-of-function mutations in *ECE1* are associated with Hirschsprung's disease (17, 27). Together, these may explain the pathogenic role of *ECE1* for HSCR (Figure 2).

In this study, we found that SNP rs169884 C > T in *ECE1* intron increases the risk of L-HSCR. Gene introns could enhance gene expression; however, the mechanism remains unclear (28). SNPs in introns could cause multiple regulatory consequences, including effects on splicing and promoter-enhancer interaction (29, 30). One recent study has reported that an intronic SNP in *BCL2* showed allele-specific enhancer activity for *BCL2* expression by affecting the transcription factor binding (31). By integrating chromatin states data, we found an enhancer signal surrounding the intronic rs169884, indicating rs169884 may have the ability to regulate *ECE1* expression. Similar as previous studies (32) which *EDNRB* and *EDN3* lead to L-HSCR as well as syndromic HSCR, we fail to explain the susceptibility for S-HSCR, which is the most common subtype in HSCR. So far, the genes responsible for the L-HSCR include *SLC6A20* (33) and *miR-618* (34). Genetic models of different genes leading to different HSCR subtypes still need to be elucidated. We recruited nearly 300 L-HSCR patients in this study; however, more patients are needed to explore more risk SNPs. Besides, environmental factors (such as toxic or drug exposure) affect birth defects should be considered to study the effect on prevention of HSCR.

Although our study is the first discovery of association between *ECE1* rs169884 and HSCR, some limitations should be noted. First, despite of the large sample size for all HSCR cases, the sample size of each subtype, especially for TCA, was still limited. Hence, we need to validate the conclusion that rs169884 is associated with an increased susceptibility of L-HSCR in larger sample size. Second, only one intronic SNP was selected in this study. The effects of other *ECE1* SNPs or SNPs in other endothelin signaling-related genes have not been explored, which might also play roles in the predisposition to HSCR. Third, although we found regulatory evidence of rs169884 in *ECE1* enhancer region in various derived neuron cells or fetal colon tissue, functional experiments are still needed to verify these results. Lastly, our study only represents the characteristic inheritance of the southern Chinese population. Further studies should focus on genotype-phenotype relationships in multi-ethnic populations.

## Conclusion

To summarize, in this HSCR case-control study from a southern Chinese population, we found an association between L-HSCR (one subtype of HSCR) and rs169884. This SNP may have the potential to regulate the expression of *ECE1*, which plays a critical role in endothelin signaling pathway during the development of enteric nervous system.

## Data Availability

The original contributions presented in the study are included in the article/Supplementary Material, further inquiries can be directed to the corresponding author/s.
